# An efficient 3D cell-based discrete fracture-matrix flow model for digitally captured fracture networks

**DOI:** 10.1007/s40789-023-00625-1

**Published:** 2023-11-01

**Authors:** Lei Sun, Mei Li, Aly Abdelaziz, Xuhai Tang, Quansheng Liu, Giovanni Grasselli

**Affiliations:** 1 Department of Civil and Mineral Engineering, University of Toronto, Toronto, ON M5S 1A4 Canada; 2 School of Civil Engineering, Wuhan University, Wuhan, 430072 China

**Keywords:** Fractured porous medium, Flow simulation, Digital image, Cell-based DFM, Finite volume method

## Abstract

Complex hydraulic fracture networks are critical for enhancing permeability in unconventional reservoirs and mining industries. However, accurately simulating the fluid flow in realistic fracture networks (compared to the statistical fracture networks) is still challenging due to the fracture complexity and computational burden. This work proposes a simple yet efficient numerical framework for the flow simulation in fractured porous media obtained by 3D high-resolution images, aiming at both computational accuracy and efficiency. The fractured rock with complex fracture geometries is numerically constructed with a cell-based discrete fracture-matrix model (DFM) having implicit fracture apertures. The flow in the complex fractured porous media (including matrix flow, fracture flow, as well as exchange flow) is simulated with a pipe-based cell-centered finite volume method. The performance of this model is validated against analytical/numerical solutions. Then a lab-scale true triaxial hydraulically fractured shale sample is reconstructed, and the fluid flow in this realistic fracture network is simulated. Results suggest that the proposed method achieves a good balance between computational efficiency and accuracy. The complex fracture networks control the fluid flow process, and the opened natural fractures behave as primary fluid pathways. Heterogeneous and anisotropic features of fluid flow are well captured with the present model.

## Highlights

- Simple yet efficient method for fluid flow simulation in digital fracture network
- Fracture representation using a cell-based DFM with implicit fracture aperture
- A pipe-based cell-centered finite volume method for fluid seepage simulation
- Achieve a good balance between computational efficiency and accuracy
- Capture heterogeneous and anisotropic fluid flow characteristics

## Introduction

Hydraulic fracturing is one of the standard techniques adopted to enhance oil and gas production in unconventional reservoirs (Jia et al. 2021; Liu et al. 2019; Osiptsov 2017; Wang et al. 2017a; Zhao et al. 2019a), as well as to improve coal seam permeability and coal caveability in mining industry (Cai et al. 2019; Huang et al. 2015; Ma et al. 2021). The existence of intrinsic discontinuities, e.g., natural fractures and bedding planes, allow for the hydraulic fracturing process to generate complex fracture networks, forming highly conductive flow channels required for economic production (Li et al. 2022; Sheng et al. 2018; Sun et al. 2022b). A better understanding of the fluid flow characteristics in complex fracture networks is essential in assessing and optimizing production.

Numerical methods are fundamental tools for studying fluid flow in fractured porous media and have been widely applied in petroleum and mining engineering (Adachi et al. 2007; Chen et al. 2022; Sun et al. 2023; Tian et al. 2021). Significant efforts have been made to develop appropriate models to simulate fluid flow in fractured porous media since the 1960s, including (1) the equivalent continuum model (ECM) (Huang et al. 2013; Sheng et al. 2020; Wu and Qin 2009); (2) dual-continuum model (DCM) (Azom and Farzam 2012; Barenblatt et al. 1960; Gerke and van Genuchten 1993); (3) discrete fracture-matrix model (DFM) (Hoteit and Firoozabadi 2008; Karimi-Fard et al. 2004; Zhao et al. 2019b) and (4) embedded discrete fracture model (EDFM) (Li and Lee 2008; Moinfar et al. 2013; Shakiba et al. 2018). In the ECM, the rock masses are assumed as isotropic and continuous media, where the fractures and matrix are represented as a single continuum based on the concept of equivalent parameters (such as equivalent permeability, porosity, etc.). The advantages of ECM are its simple data requirement and computational efficiency; however, the distinct hydraulic property differences of rock matrix and fractures are ignored, and the calculation of an equivalent parameter, as well as the interaction between matrix and fractures are still questionable (Huang et al. 2013). The DCM further subdivides the reservoir into two interacting media, i.e., the intact rock matrix and fracture network, with different fluid storage and conductivity characteristics. It is assumed that the fractures are uniformly embedded in the matrix, and the interaction between the matrix and the fractures is represented by transfer functions. However, the DCM hardly considers any actual geological descriptions (e.g., location, connection) or characteristics (e.g., aperture) of the fractures, especially for sparse or poorly connected fracture networks (Bai 1999). To accurately represent the fractures, the DFM was developed, where the fractures are represented by objects of codimension one (i.e., surfaces for three-dimensional (3D)), and unstructured matrix meshes are generated to conform to the fractures’ geometry such that fractures lie at the interfaces between matrix cells. The DFM provides a more realistic and physics-based representation of the fractured reservoirs, however, generating 3D unstructured meshing is quite complicated for complex fracture networks (Karimi-Fard et al. 2004), which also makes the fluid flow calculation for high-resolution fractures often computationally expensive. Furthermore, EDFM is another alternative where fractures are embedded within non-confirming matrix blocks and the fracture-matrix interaction is achieved by identifying the connections between fracture cells and matrix cells. It honors the accuracy of DFMs by explicitly representing the fractures while maintaining the efficiency offered by nonconforming meshes, as in DCM. However, EDFM still has difficulty in handling the cases in which the fracture permeability lies below that of matrix, and multiple fracture interaction in one coarse cell (Rao et al. 2020; Ţene et al. 2017).

Although extensive work has been conducted to simulate fluid flow in the fractured medium, predicting the flow through a real fractured system remains challenging, due to (i) lack of realistic representation of the complexity of the fracture geometry, and (ii) lack of information pertaining to the fracture properties (Adachi et al. 2007; Sheng et al. 2019; Tokan-Lawal et al. 2015). In most numerical studies (Ebigbo et al. 2016; Fumagalli et al. 2016; Hui et al. 2018; Lang et al. 2014; Thomas et al. 2020), stochastic methods are generally used to generate fracture networks, where a series of physical or geometrical statistical parameters (including location, orientation, aperture, and length) are integrated from multiple data sources (e.g., well logs, core analysis, seismic data, tectonic history, production history, etc.) (Gilbert et al. 2004; Tian et al. 2017; Xu et al. 2015). However, these stochastic fracture networks rely heavily on geo-statistical analysis and thus have fundamentally different topological properties compared to the actual subsurface fractures (e.g., heterogeneous apertures, anisotropic fracture orientations, nonplanar geometries, fracture intersections) (Frash et al. 2019; Thomas et al. 2020).

Recent advances in fracture diagnostic technology (e.g., x-ray computer tomography scans, magnetic resonance imaging, photogrammetry, and ultrasound) have brought new insights to the complexity of the fracture geometries, and allowed more reliable fracture information (Abdelaziz et al. 2023; Li et al. 2022; Ramandi et al. 2017; Shi et al. 2020; Wang et al. 2021; Xiong et al. 2021). These sophisticated geometric digital fracture representations presents a better quantitative evaluation of the fracture geometrical features; however, challenges remain in representing the fractures and simulating the fluid flow process in the numerical simulations (Wu 2021, 2022). For example, the spatial resolution of the geometric digital fractures (at voxel level) are several orders of magnitude smaller than the simulation domain. In addition, fractures always have irregular geometry and intricate topology. One widely used method to reconstruct the fracture network is the statistical Discrete Fracture Network models (DFN) where the geometrical statistics of the fracture are evaluated from digital images (Jing et al. 2020; Sun et al. 2022a; Voorn et al. 2015). However, discretization of the DFN prior to the numerical simulation is labor-intensive and time-consuming, especially when several fractures intersect with each other in an irregular connectivity pattern. In addition, DFN models are commonly comprised of planar fractures that are circular (or rectangular) in shape, while in-situ fractures have irregular shapes and varying openings. Another method is to perform numerical simulation directly on the digital image volume, where each image voxel is simply treated as an interpolation element while fractures are represented as high-permeability blocks (Mostaghimi et al. 2013; Wang et al. 2017b; Xiong et al. 2021). Despite the considerable advantage that direct simulations provide in accurately depicting the complexity of the fracture network, they are computationally demanding especially when the image dataset is large (for example, the sample in Sect. 3 has approximately 2000^3^ voxels).

This paper proposes a simple yet efficient systematic framework to simulate the fluid flow in a 3D realistic fractured porous medium. The main idea of the proposed method is to achieve a good balance between computational efficiency, flexibility, and accuracy. A cell-based DFM is developed to capture the complex fracture geometry from the digital fracture dataset, where the fractures are virtually represented in a set of cells with implicit fracture aperture. The fracture geometry, connectivity, and topological properties (e.g., nonplanar shape and variable aperture) derived from the real fracture network are well preserved in the numerical model. A cell-centered finite volume method is then used to simulate the fluid flow in the reconstructed fractured porous medium with a virtual pipe network model where the different transmissibility values are assigned between matrix-matrix, fracture-matrix, and fracture-fracture connections. More specifically, the varying aperture and the fracture connectivity can well reproduce the heterogeneity and the anisotropic nature of the fractured rock masses. The accuracy and computational efficiency of the proposed model are demonstrated against analytical/numerical results, and the mesh sensitivity is also discussed. Finally, a lab-scale case study is performed to show a potential application of the model in a hydraulically fractured reservoir with natural fractures, where the fracture network is reconstructed from a true triaxial hydraulically fractured shale sample using the serial-section reconstruction approach.

This paper is organized as follows: Sect. 2 introduces the fundamental framework of the fracture network reconstruction as well as the fluid flow simulation in the numerical models. Validation tests are presented to demonstrate the accuracy and applicability of the proposed method. In Sect. 3, 3D map of the fracture complexity of a hydraulically fractured shale sample at micron-scale spatial resolution is introduced. Then the fluid flow in the fractured shale sample is modelled. Discussion and conclusion are outlined in Sects. 4 and 5, respectively.

## Numerical framework for fluid flow simulation in fractured porous medium

This section introduces an efficient cell-based discrete fracture-matrix model (DFM) model, programmed in C language, capable of conducting fluid flow simulation of a complex fracture network digitally reconstructed at a high spatial resolution. Fluid flow, including matrix flow, fracture flow, and fluid exchange between fracture-matrix, are performed with a cell-centered finite volume scheme with equivalent pipe network model.

### Cell-based DFM model for fracture network representation

**Fig. 1:**
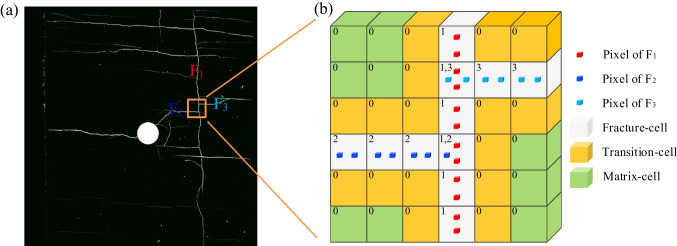
**a** A digital image of fracture networks (Li et al. 2022); **b** Schematic of the cell-based DFM based on the digital data. Three types of cells are defined. Fracture-cells are labelled with different phase (e.g., *φ* = 1, 2, 3) according to the fracture set they represent while the transition- and matrix-cells are labelled as *φ* = 0

With the aid of advanced fracture diagnostic technology, geometries of complex rock masses are measured and recorded precisely as high-quality 3D digital image volumes (Fig. 1a). Importing this geometric information, expressed at the pixel level, provides more realistic and accurate geometrical representation within the model.

A cell-based DFM is proposed herein to represent the fracture networks based on the imported digital images (e.g., point cloud dataset), to achieve a balance between the accuracy and computational efficiency. As shown in Fig. 1b, in the cell-based DFM, the simulation domain is first discretized into hexahedral grids. The resolution of the user-determined grid is independent of the pixel size of the image and the spatial distribution of the macro features. Then, a voxelization process is performed to convert the discrete geometry of fractures (represented by voxel clouds) to discrete cells. According to the relative location of the fracture voxel, three kinds of cells are defined (Fig. 1b): the cell containing fractures is the fracture-cell, the cell neighboring the fracture-cell is transition-cell, and the remaining cell is matrix-cell. A variable “phase” (*φ*) is defined on the fracture-cells, i.e., which contains fracture pixel, and are flagged with the fracture set number, otherwise are set as *φ* = 0. More specifically, the fracture-cells containing multiple fractures are tagged with all the fracture sets that pass through it, to show the fracture connectivity. It should be noted that the fracture-cells here are not the exact fracture, but rather a rough representation of the fracture location and connectivity of the fracture network obtained from the digital data. The finer the mesh size, the more accurate the fracture representation. When the cell size is the same as that of the image resolution, this model degrades to the direct simulation method (Mostaghimi et al. 2013; Wang et al. 2017b), which is computationally expensive.

For flow simulations, it is important that the constructed fracture network model captures the heterogeneous spatial distribution of apertures. The fracture aperture is hard to be explicitly represented because it is often much thinner than the model grid size. In the present method, the fracture aperture is implicitly represented by the equivalent fracture aperture based on the principle of average local permeability (Eq. (1)). For a fracture-cell passed by *j*-th fracture, the equivalent fracture aperture (*a*_*e,j*_) in this cell could be given as:

1 $$a_{e,j}^{3} = \frac{1}{np}\sum\limits_{i = 0}^{np} {a_{i,j}^{3} }$$

where *a*_*i,j*_ is the aperture of *i*-th fracture pixel of the *j*-th fracture in the cell, and *np* is the total number of the fracture pixels in the *j*-th fracture in the cell. Each cell could have a different local aperture corresponding to the fracture heterogeneity. Particularly, for intersected fractures in one cell, since the fracture and cell are labeled, apertures of each fracture could be calculated separately. The specific aperture variation preserves the features of heterogeneity and anisotropy of the local permeability. This approach also allows the treatment of the fractures as a fluid channel with equivalent permeability in a coarse mesh, so that special treatment for explicitly tracing the actual 3D fracture geometry is avoided.

In the cell-based DFM method, the mesh size is independent of the image resolution, where the system degree of freedoms greatly decreases when compared to the direct simulation method (Mostaghimi et al. 2013; Wang et al. 2017b). In addition, the proposed method allows for the effortless and undemanding pre-processing of importing the digital image (voxel cloud) enabling the reconstruction of the complex fracture geometry (e.g., various apertures with arbitrary shape) into a cell-based fracture. This process eliminates burdensome tasks, like surface tracking and statistical data analysis, that are associated with stochastic methods (Jing et al. 2020; Voorn et al. 2015). The specific aperture and labelled fracture connectivity within each fracture-cell can well represent the heterogeneity and the anisotropy feature of the local permeability, resulting in a more realistic fluid flow calculation. Thus, the proposed method achieves a balance between accuracy and computational efficiency, as further discussed in Sect. 4.

### Fluid flow simulation in fractured porous media

The reconstructed model is then utilized to perform flow simulations using a cell-centered finite volume approach with the aid of an equivalent pipe model, as its computational efficiency. Both matrix pores and fractures form preferential paths for fluid flow, and the seepage within a rock mass comprises of three main components (Yan et al. 2018): fluid flow in fractures (fracture flow), fluid flow in rock matrix (matrix flow), and the fluid exchange between fractures and rock matrix (exchange flow).

**Fig. 2:**
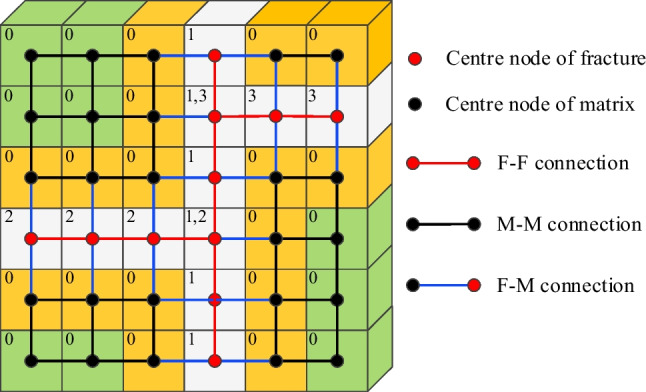
The fluid flow between two cells is conceptualized as 1D equivalent pipe flow. Three types of pipes are defined: connection between fracture–fracture (F–F), matrix–matrix (M–M) and fracture-matrix (F–M)

Each cell is treated as the corresponding control volume, and the fluid information (e.g., fluid pressure, flow flux, and pore volume) is stored at the center of the cell. These spatially interconnected pores and fractures are conceptualized as interconnected pipes (Fig. 2). The numerical fluid flow in rock matrix (a low permeability medium), fracture networks (a high permeability medium), and fluid exchange between fracture and matrix are simulated by virtual permeable pipe networks with equivalent flow parameters. This approach allows the treatment of the fractures as a simple one-dimensional (1D) fluid channel with equivalent permeability, hence, special treatment of the actual fracture geometry in DFM method is avoided, which is difficult to obtain in the point cloud data.

The continuity equation for the fluid flow in fractured porous medium can be given as:

2 $$\frac{\partial \rho }{{\partial t}} + \nabla \cdot (\rho {q}) = q_{s}$$

where *t* is the time, *ρ* is the fluid density, *q* is the velocity vector,* q*_*s*_ is sink/source term.

Darcy’s law is assumed to be applicable for the flow in both fractures and matrix, where the pipe flow has the following unified form (Ren et al. 2016; Zimmerman and Bodvarsson 1996):

3 $$q_{ij} = - T_{ij} \Delta P$$

where *q*_*ij*_ is the flow rate between cells *i* and *j*. *T*_*ij*_ is the transmissibility of the pipe *ij*. *P* = *p* − * ρgh* is the total pressure at pipe ends, where *p* is the fluid pressure, *h* is the elevation head, *g* =  − 9.8 m/s^2^ is the gravity acceleration. Thus, the challenging part is calculating the transmissibility for fracture-fracture, fracture-matrix, and matrix–matrix entities, as well as their connections.

#### M–M connection

**Fig. 3:**
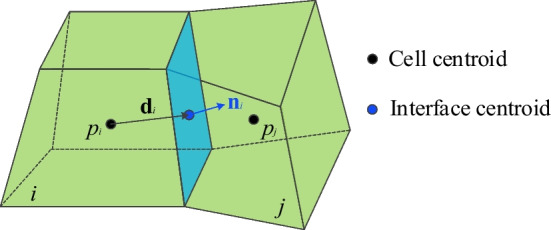
Schematic of two-point flux-approximation for transmissibility between M–M connections

For the matrix flow (which constitutes both connection between matrix–matrix and matrix-transition cells), the widely acceptable two-point flux-approximation (TPFA) (Fumagalli et al. 2016; Karimi-Fard et al. 2004; Zhao et al. 2019b) is used to evaluate the fluid flow transmissibility between two cells (matrix grids) (Fig. 3). The transmissibility (*T*_*ij*_) is the harmonic average of the half transmissibilities from the centroid of the cell to the interface, defined as:

4 $$T_{ij} = \frac{{T_{i} T_{j} }}{{T_{i} + T_{j} }}$$

5 $$T_{i} = \frac{{k_{i} A_{ij} }}{{\mu \left| {{d}_{i} } \right|^{2} }}{d}_{i} \cdot {n}_{i} ,\quad T_{j} = \frac{{k_{j} A_{ij} }}{{\mu \left| {{d}_{j} } \right|^{2} }}{d}_{j} \cdot {n}_{j}$$

where *T*_*i*_ and *T*_*j*_ are the half transmissibilities of cell *i* and *j*, respectively. *k*_*i*_ and* k*_*j*_ are the permeability in cell *i* and *j*, respectively. *μ* is fluid viscosity. *A*_*ij*_ is the area of the interface between the two cells, *d*_*i*_ and *d*_*j*_ are the vectors from centroids of cell *i* and *j* to the centroid of the interface, respectively. *n*_*i*_ and *n*_*j*_ are the outward unit normal vector of the interface as a part of cell *i* and *j*, respectively.

#### F–F connection

**Fig. 4:**
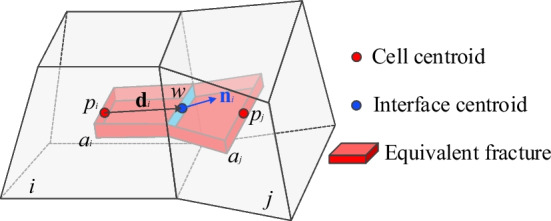
Schematic of transmissibility of F–F connections

For the fluid flow in fracture-fracture connection, the fracture flow is represented by pipes connecting the two fracture-cells (Fig. 4). Since the accurate fracture location within the cell is unknown, we assume that the fracture segment passes the interface center of the two connected cells. A similar idea to the M-M connection can be adopted and the transmissibility between F-F connection can be calculated as:

6 $$T_{ij} = \frac{{T_{i} T_{j} }}{{T_{i} + T_{j} }}$$

7 $$T_{i} = \frac{{A_{ij} }}{{\left| {{\mathbf{d}}_{i} } \right|^{2} }}k_{fi} {d}_{i} \cdot {n}_{i} ,\quad T_{j} = \frac{{A_{ij} }}{{\left| {{\mathbf{d}}_{j} } \right|^{2} }}k_{fj} {\mathbf{d}}_{j} \cdot {\mathbf{n}}_{j}$$

where the definition of **d**_*i*_ and **d**_*j*_, **n**_*i*_ and **n**_*j*_ are the same as those in M-M condition. However, since the fractures are implicitly represented with varying aperture, fracture permeability *k* and interface area *A* should be redefined.

For each pipe element of a fracture-cell, we assume a rectangular conduit (Fig. 4), where the conduit height is equal to the corresponding fracture aperture, the conduit width is determined by the equivalent flow seepage width (cell width), and the conduit length is the distance between the two end-nodes. The parallel plate model is then applied, where the permeability and interface area of the two fracture segments are defined as (Zimmerman and Bodvarsson 1996):

8 $$k = - \frac{{a^{2} }}{12\mu }$$

9 $$A_{f} = aw$$

where *a* is the equivalent crack aperture (Sect. 2.1), *w* is the cell width.

In this paper, fracture-cells are labeled with the fracture index. For the flow channel between fracture-cells with the same index, the specific fracture aperture for each fracture is used. In case that fracture-cells pass multiple fractures, the fracture aperture may vary between different fracture sets. For these intersection elements, the fluid flow in each fracture set can be calculated separately with different permeability (e.g., for an intersection element in Fig. 2, different fracture aperture could be assigned to connected elements indexed with 1–1 and 1–2). Thus, the heterogeneity and the anisotropy of the local permeability tensor on the fracture are honored within the simulation.

#### F–M connection

**Fig. 5:**
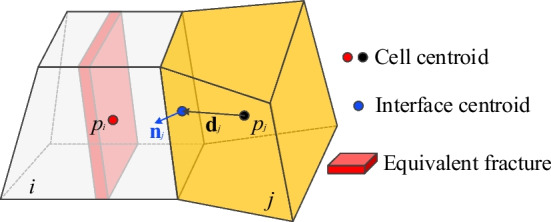
Transmissibility of F–M connection

Pressure gradients between the rock matrix and fractures will induce fluid exchange, referred to as exchange flow (Fig. 5), where the transmissibility can be modified as:

10 $$T_{ij} = \frac{{T_{i} T_{j} }}{{T_{i} + T_{j} }}$$

where the half transmissibility of the transition element is the same as that of the matrix cell. However, the half transmissibility of fracture element is represented using the leak-off model (Yarushina et al. 2013):

11 $$T_{i} = k_{c}$$

where *k*_*c*_ is the fluid exchange coefficient between the fracture and rock matrix.

#### Pressure update

The total flow rate of each cell can be calculated as the summation of the flow rates associated with all connected channels. The fluid pressure, *p*, at each cell can be updated as (Liu and Sun 2019; Yan et al. 2018):

12 $$p = p_{0} + K_{\text{w}} Q\frac{\Delta t}{V}$$

where *p*_*0*_ is the pressure at the previous time step, *K*_w_ is the bulk modulus of the fluid, *Q* is the total flow rate, ∆*t* is the time increment, *V* is the hydraulic volume of the cell, and the hydraulic volume of the matrix cell (*V*_M_), transition cell (*V*_T_), and fracture cell (*V*_F_) is calculated as:

13 $$V_{\text{M}} = V_{\text{T}} = \phi V_{\text{e}}$$

14 $$V_{\text{F}} = \delta V_{\text{e}} /h$$

where *V*_e_ is the cell volume, *ϕ* is the porosity, *δ* is the fracture aperture, and *h* is the mesh size.

The explicit time integration scheme is applied on cell-by-cell, which eliminates the need for Jacobian matrices, and overcomes the computational challenge associated with large scale problems.

#### Initial condition and boundary conditions

The initial condition can be given by:

15 $${u}(t = 0) = u_{0}$$

where *u* is variables (e.g., fluid pressure and fluid flux). *u*_0_ is a known function of time or a prescribed value of variable *u* at the initial time.

Two types of boundary conditions are considered in this model:

(i) Dirichlet boundary condition:

16 $${u}\left| {_{\Gamma } } \right. = u_{\text{s}}$$

(ii) Neumann boundary condition:

17 $$\frac{\partial u}{{\partial {n}}}\left| {_{\Gamma } } \right. = f_{\text{s}}$$

where *u*_s_ is the prescribed value of variable *u* at the boundary Γ. *n* denotes the normal vector to the boundary, and *f*_s_ is a given scalar function at the boundary Γ.

**Fig. 6:**
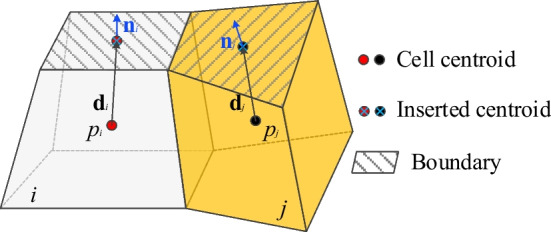
Schematic of boundary treatment. Boundary centroids are inserted at the boundary surfaces

Since the pressure is stored at the center of the cells, while the boundary is always assigned on the surface of the system, a set of boundary center points are inserted onto the boundary surface to facilitate the numerical calculation (Fig. 6). Similarly, the transmissibility between the boundary node and cell center can be given as:

18 $$T_{bm} = \frac{{A_{ij} }}{{\left| {{d}_{i} } \right|^{2} }}K_{fi} {d}_{i} \cdot {n}_{i}$$

19 $$T_{bf} = \frac{{\delta^{3} h}}{{12\mu \left| {{d}_{j} } \right|^{2} }}{d}_{j} \cdot {n}_{j}$$

where *d*_*i*_ and *d*_*j*_ are the vectors from centroids of cell *i* and *j* to the centroid of the boundary surface, respectively. *n*_*i*_ and *n*_*j*_ are the outward unit normal vector of the boundary surface of cell *i* and *j*, respectively.

### Validation tests

In this section, numerical tests are conducted to validate the ability of the proposed model to simulate fluid flow in fractured porous media. Tests 1 and 2 are used to validate the fluid flow in rock matrix and fracture, respectively, by comparing it with analytical solutions. Test 3 is then used to validate the fluid flow in both fracture and rock matrix against numerical results, which is simulated using the combined finite-discrete element method (FDEM) with a DFM model.

#### Fluid flow in rock matrix

**Fig. 7:**
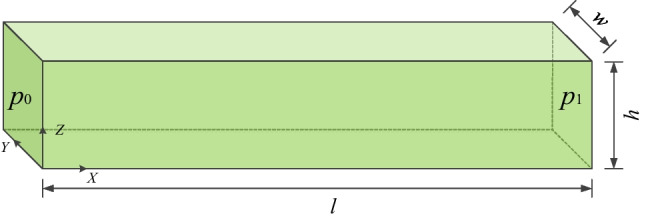
Fluid flow in an intact rock sample

The fluid transfer in an intact rock is validated against the analytical solutions (Carslaw and Jaeger 1959). The rock sample is 1.0 m × 0.2 m × 0.2 m (Fig. 7), with the porosity of *ϕ* = 0.1 and permeability of *k* = 1 × 10^−13^ m^2^. The rock is assumed to be fully saturated, and the initial pore pressure is set to be zero. Prescribed pressure boundaries are assigned at the left and right sides of *p*_0_ = 100 and *p*_1_ = 0 kPa, respectively, and the other boundaries are impervious. The fluid parameters adopted in the following tests are: fluid density *ρ* = 1000 kg/m^3^, bulk modulus *K*_w_ = 2.0 GPa, and viscosity μ = 1 × 10^−3^ Pa s, unless otherwise stated.

The analytical solution of the hydraulic pressure distribution during the fluid transfer process is (Carslaw and Jaeger 1959; Yan et al. 2018):

20 $$p(x,t) = p_{0} + \frac{x}{l}(p_{1} - p_{0} ) + \frac{2}{\uppi }\sum\limits_{i = 1}^{\infty } {e^{{ - T\uppi^{2} i^{2} /l^{2} }} (\frac{{p_{1} \cos (ix) - p_{0} }}{i})} \sin \frac{ix\uppi }{l}$$

21 $$T = ktM/\mu$$

where *x* is the distance to the left boundary, *l* is the length, *T* is non-dimensional time. *M* = *K*_w_/*ϕ* is the Biot modulus.

**Fig. 8:**
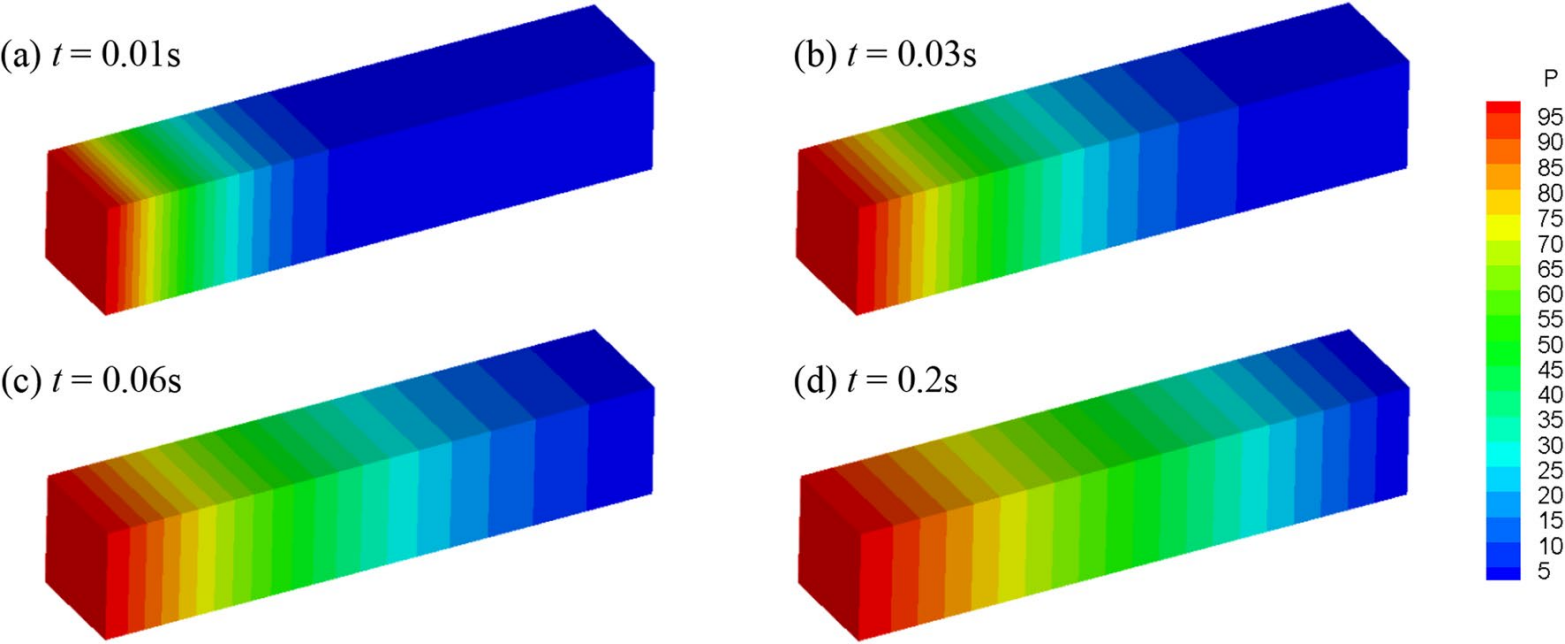
Fluid pressure distribution (kPa) at *t* = 0.01 s, 0.03 s, 0.06 s and 0.2 s

**Fig. 9:**
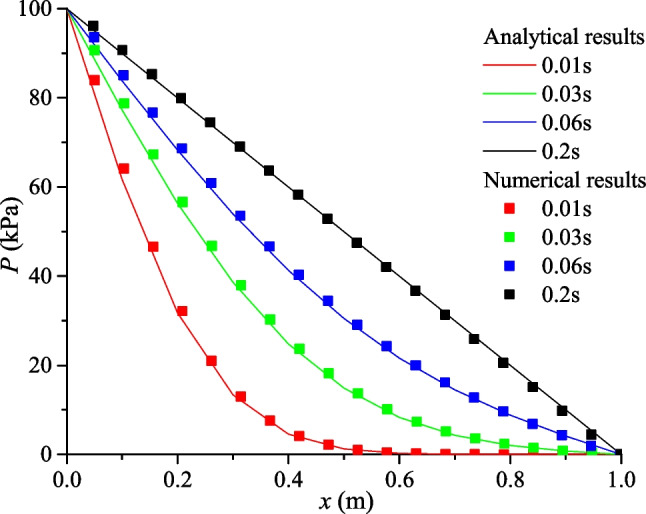
Comparison of the analytical and numerical results of the fluid pressure distribution at different times

The fluid pressure evolution in the rock mass is shown in Fig. 8. Due to the pressure gradient, fluid flows from the left side to the right side, and the fluid pressure increases with time. A comparison between the analytical and numerical solution is presented in Fig. 9, where a good agreement can be observed. In addition, when the fluid flow reaches steady state (about 0.2 s), the fluid pressure obeys a linear distribution. The monitored fluid flux at the steady state is approximately 1.003 × 10^−5^ m/s, which is close to the analytical value (1 × 10^−5^ m/s) calculated according to Darcy’s law:

22 $$q = \frac{k}{\mu L}\Delta p$$

where ∆*p* is the pressure difference across the sample and *l* is the sample length.

#### Fluid flow in a single fracture

**Fig. 10:**
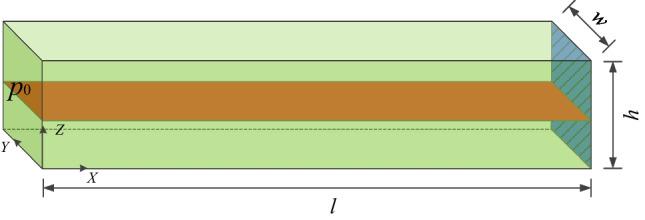
Transient fluid flow in a rock sample with single fracture

This test validates the fluid flow in a single fracture. The geometry of the rock sample is the same as that in Sect. 2.3.1, except for a single fracture inserted at the center of the sample with an aperture of *a* = 1 × 10^−3^ m (Fig. 10). Constant fluid pressure *p*_0_ = 100 kPa is applied to the left boundary, while the right end of sample, as well as other boundaries are impervious.

When only fracture flow is considered, i.e., the matrix permeability is 0 and the exchange flow is ignored, the analytical solution of this test is (Carslaw and Jaeger 1959; Sun et al. 2019):

23 $$\frac{{p(x,t)}}{{p_{0} }} = 1 + \frac{4}{\uppi }\sum\limits_{{n = 0}}^{\infty } {e^{{ - (2n + 1)^{2} T\uppi ^{2} /4}} \left( {\frac{{( - 1)^{{n + 1}} }}{{2n + 1}}} \right)} \cos \left( {\frac{{(2n + 1)(l - x)\uppi }}{{2l}}} \right)$$

24 $$T = K_{\text{w}} \frac{{a^{2} t}}{{12\mu l^{2} }}$$

where *x* is the distance to the left side; and *p* is the fluid pressure. *T* is non-dimensional time.

**Fig. 11:**
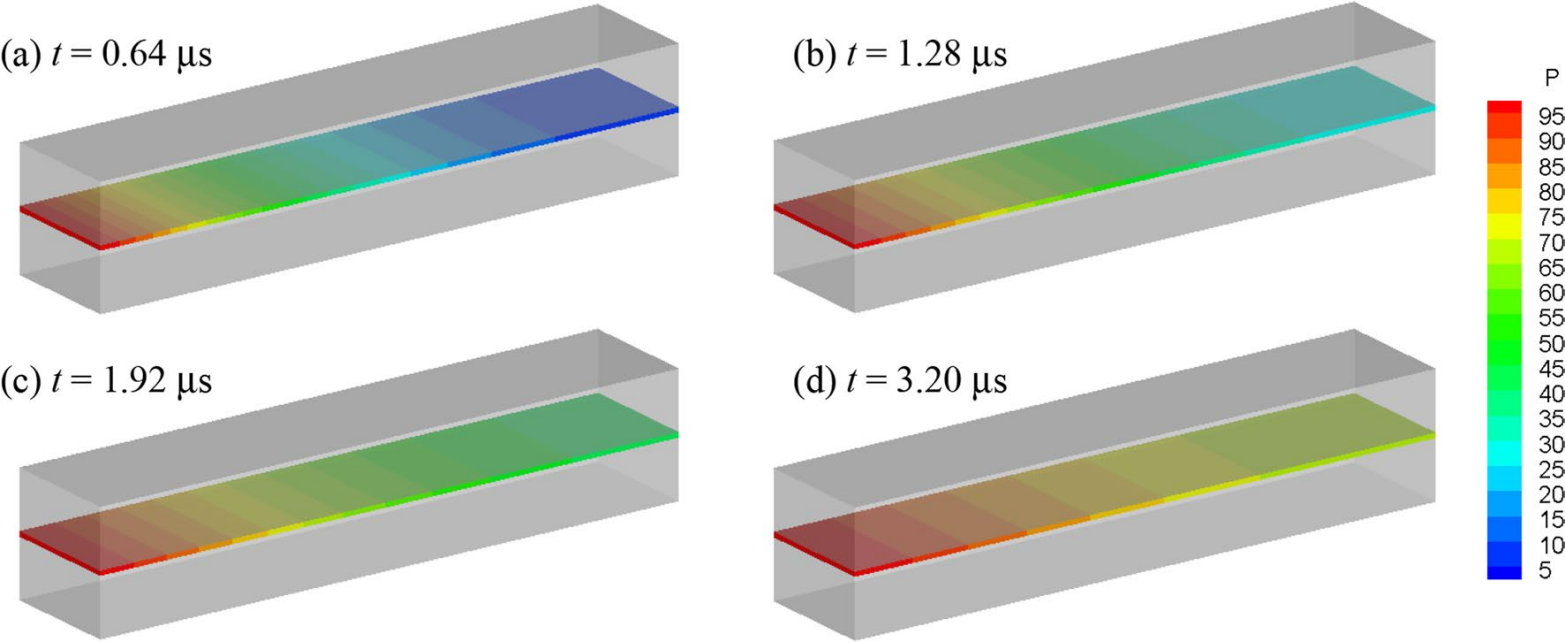
Fluid pressure distribution (kPa) at *t* = 0.64 µs, 1.28 µs, 1.92 µs and 3.20 µs

**Fig. 12:**
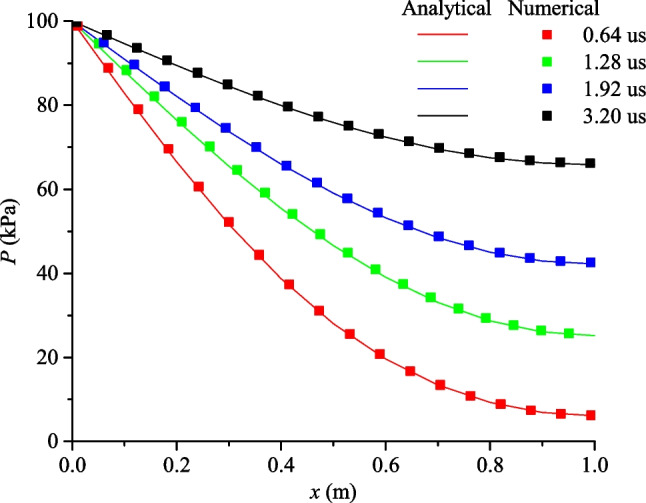
Comparison of the analytical and numerical results of the fluid pressure distribution in a rock sample with a fracture at different times

Fluid pressure distribution at different times is shown in Fig. 11 which depicts an increase in fluid pressure along the *x*-direction with increase in time. A nonlinear decrease of fluid pressure along the fracture can be observed. A comparison between the analytical and numerical solution is presented in Fig. 12, where the numerical results agree well with the analytical solution.

**Fig. 13:**
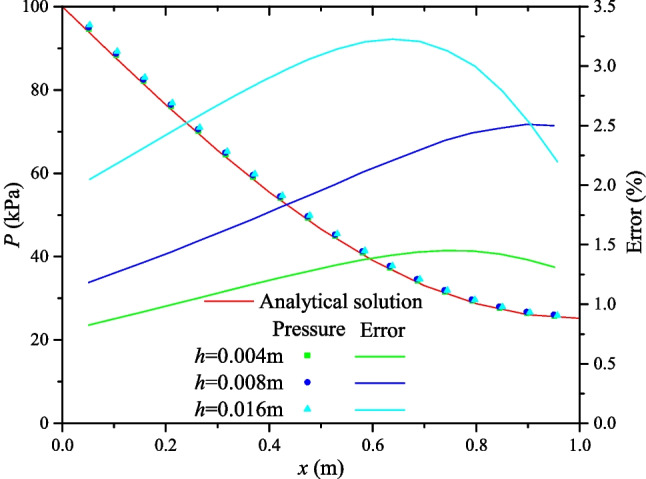
Fluid pressure and error for three different mesh size (*h* = 4 mm, 8 mm, and 16 mm) at *t* = 1.28 µs

One important advantage of the proposed approach is that the fracture information is represented implicitly, thus it does not require a very fine grid to perform the simulation. A mesh size sensitivity study is carried out, with mesh size of ℎ = 0.016, 0.008, and 0.004 m. Figure 13 shows the pore pressure distribution of the numerical result at *t* = 1.28 µs for the different mesh sizes. Overall, the pore pressure distribution calculated for the various mesh sizes matches well the analytical solution. In addition, the relative error between the analytical solution and the numerical solution for the three sets of mesh sizes shows that the error decreases with decreasing mesh size. In other words, the smaller the mesh size, the more accurate the numerical solution. However, the errors of pore pressure distribution at each nodal point are all less than 0.6% for the three element sizes, which clearly illustrates that the element size has minimal effect on the numerical results.

#### Mixed fracture-matrix flow

**Fig. 14:**
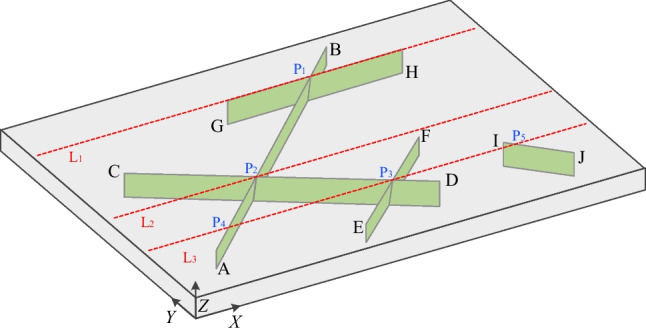
Geometric model of a fracture-imbedded porous medium (after Yan et al. 2022). The fractures are shown in green, and the monitoring lines are shown in red. Points P denote intersection points

In this test, a benchmark test (Yan et al. 2022) of a fractured porous media seepage is investigated, where both fracture seepage, matrix seepage, and fluid exchange are considered. The rock sample is 40 m × 30 m × 2 m (Fig. 14) and contains 5 imbedded fractures parallel to the *Z*-axis (the specific coordinates are shown in Table 1). The initial pressure within the model is 0 MPa. The pressure at the left (*X* = 0) and right (*X* = 40) boundary is fixed at 100 kPa and 0 kPa, respectively, and the other boundaries are impervious. The specific parameters used in the model are as follows: the permeability of the matrix *k* = 5 × 10^−13^ m^2^, the porosity of the matrix *ϕ* = 0.1, the hydraulic aperture for the fracture is *a* = 1 mm, and the fluid exchange coefficient *k*_c_ = 2 × 10^−8^ m/Pa s (Yan et al. 2022).

**Table 1:** The coordinates of the fracture ends

Points	A	C	E	G	I
Coordinates	(5.0, 5.0, 2.0)	(5.0, 19.0, 2.0)	(17.0, 3.0, 2.0)	(17.5, 25.0, 2.0)	(32.5, 9.0, 2.0)

Points	B	D	F	H	J
Coordinates	(27.5, 27.5, 2.0)	(25.0, 6.0, 2.0)	(27.0, 12.5, 2.0)	(32.5, 25.0, 2.0)	(36.0, 5.0, 2.0)

**Fig. 15:**
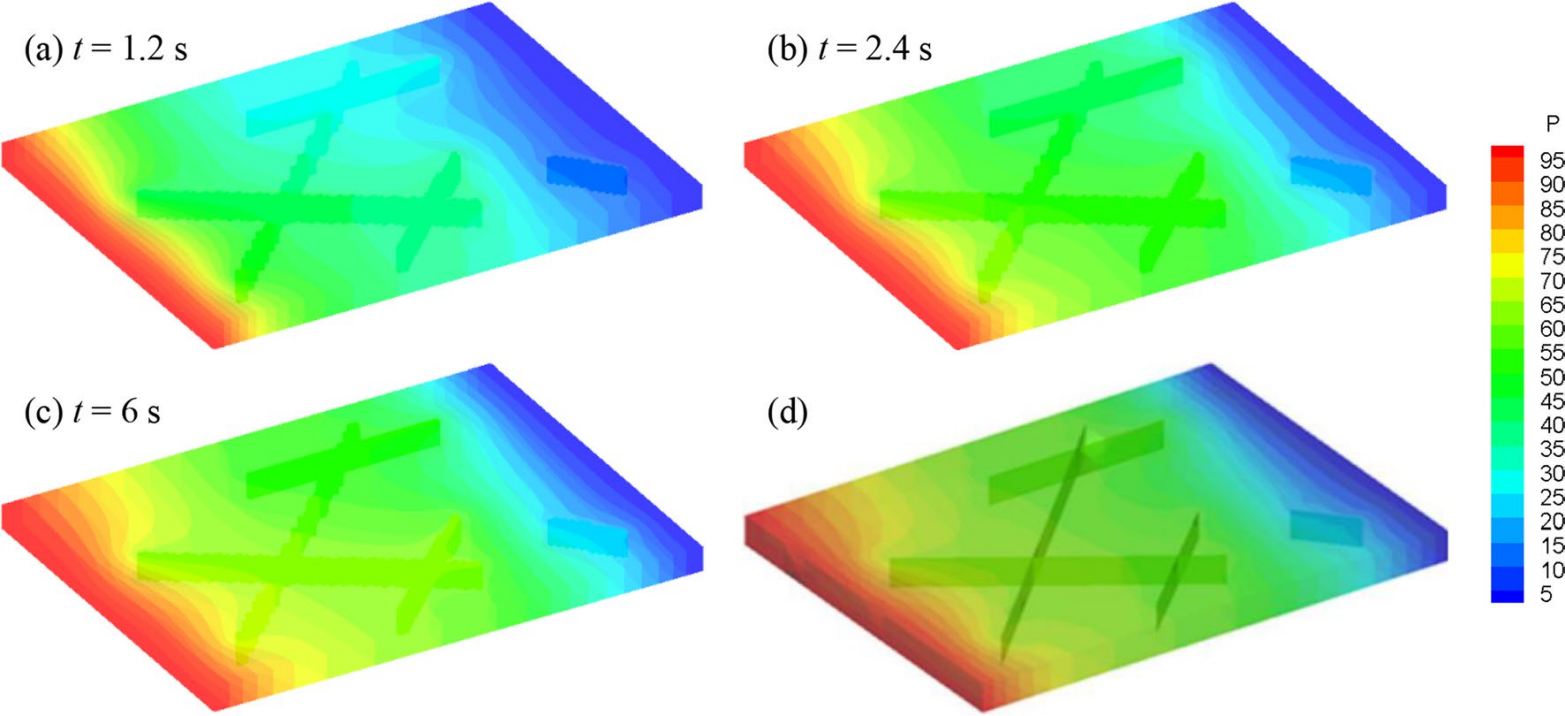
Simulation results: **a**–**c** The pore pressure distribution (kPa) obtained by the model in this paper; **d** The pore pressure distribution at steady-state obtained by Yan’s model (Yan et al. 2022)

The fluid flows from the left to the right side, and the pore pressure distribution at different times is shown in Fig. 15. Compared with the matrix, cracks have much higher permeability. Therefore, the buildup of the pressure around crack tips A and C appears to be slower than that of the surrounding matrix when the fluid exchange occurs between the matrix and fracture. On the other hand, pressure around crack tips H, F, and D is larger than the pore pressure of the surrounding matrix. It is clear that the fracture with higher permeability serves as the main fluid flow channel, showing anisotropic fluid flow. Meanwhile, the inclined isolated fracture blocks the fluid flows through the fracture (e.g., IJ), while fractures parallel to the pressure gradient accelerate the fluid flow along the fracture (e.g., GH). With the continued transmission of pore pressure, the pore pressure in the medium domain gradually increases until it stabilizes (*t* = 6 s, Fig. 15c). The fluid pressure in the fractured porous media is significantly affected by the crack distribution. Compared to the existing results (Fig. 15d, which is simulated by the FDEM with a DFM model, after Yan et al. 2022), the simulation results at steady-state shows a good agreement.

**Fig. 16:**
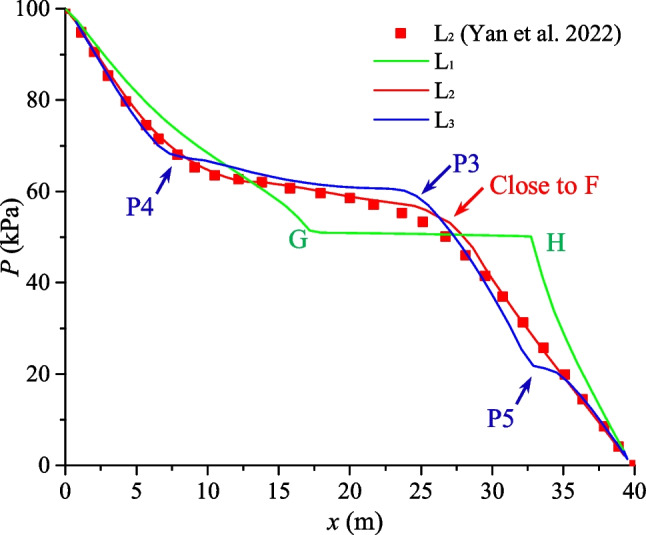
Fluid pressure distribution along the monitoring lines. The numerical results of L_2_ in the proposed model is also compared with existing results (Yan et al. 2022)

Furthermore, three monitoring lines (Red lines in Fig. 14) are set to quantitatively show the pressure distribution at the steady-state (Fig. 16). In monitoring line L_1_, which passes the horizontal fracture (GH), a step with low-pressure gradient can be observed. The reason can be explained that the permeability of the fracture is much larger than the permeability of the rock matrix, requiring a lower pressure gradient for the same fluid flux at the steady state. We can also observe several inflection points in monitoring lines L_2_ and L_3_, which correspond to the intersection points of fractures. Since the permeability of the fractures is much greater than that of the rock matrix, these cracks become channels for preferential fluid flow. As a result, the flow and pressure redistribution within the fracture network and the rock matrix are significantly affected by the surrounding fractures. Furthermore, the results of monitoring line L_2_ in the middle of the model (cross one intersection point) are in good agreement with the existing results of Yan et al. 2022, further verifying the applicability of the proposed model for solving fluid flow in complex fractured porous media.

## Fluid flow simulation of a digitally captured fracture network

In this section, a lab-scale true triaxial hydraulically fractured shale sample is digitally reconstructed, and the proposed numerical model is utilized to simulate the fluid flow behavior in this realistic fracture network.

### Fracture mapping of a hydraulically fractured rock sample

**Fig. 17:**
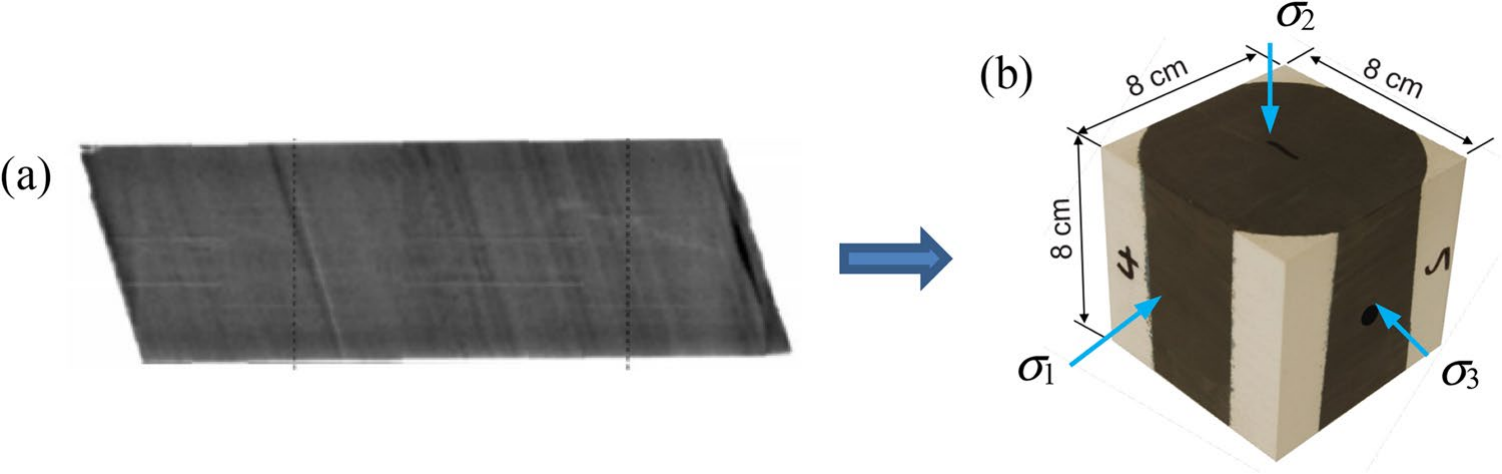
**a** The Montney shale core and **b** Geometry of the shale cube rock sample. (The black part is Montney shale core, and the white part is Gypsum cement which encapsulates the core to form a cube sample.)

A laboratory hydraulic fracturing test under true triaxial stress conditions (TTT-HF) was conducted, and the 3D fracture complexity was then mapped. The test sample (80 mm × 80 mm × 80 mm, Fig. 17) was shale reservoir rock obtained from a depth of approximately 2000 m in the Montney formation (Western Canadian Sedimentary Basin). A 6.4 mm diameter hole was drilled at the center of the cube face to a depth of approximately 44.5 mm to act as an open well during the hydraulic fracturing test (Abdelaziz et al. 2019).

**Fig. 18:**
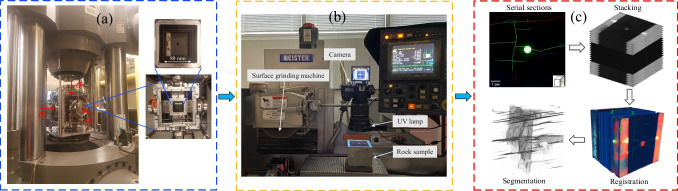
The procedure of the fracture mapping of a hydraulically fractured rock sample. **a** TTT-HF experiment **b** Surface grinding and photographing, and **c** Serial-section reconstruction

The TTT-HF experiment was conducted at the Rock Fracture Dynamics Facility at the University of Toronto (Fig. 18a) (Abdelaziz et al. 2019; Lombos et al. 2013). The effective stress state applied to the center of the cube in the system are *σ*_1_ = 48.2 MPa, *σ*_2_ = 34.2 MPa, and *σ*_3_ = 27.8 MPa (Fig. 17b), to mimic those of the reservoir. The mini-well was oriented along the minimum principal stress direction, and designed as a single stage open-hole which is a common completions practice within Montney formation. Fluid, more specifically slick water, was injected at constant flow rate (7 mL/min) once the reservoir stress state was reached. Specifics and mechanical response of the TTT-HF experiment can be found in Abdelaziz 2023.

After the TTT-HF experiment, the serial-section reconstruction method was used to three-dimensionally map the post-test fracture complexity (Figs. 18b, c), at micron-scale resolution (39 μm × 39 μm × 50 μm). A complex fracture network, as opposed to a single planar fracture, was observed in the shale sample due to the influence of pre-existing fractures and material fabric during the hydraulic fracture propagation process. Details pertaining to the fracture mapping using the serial-section reconstruction method can be found in Li et al. 2022.

**Fig. 19:**
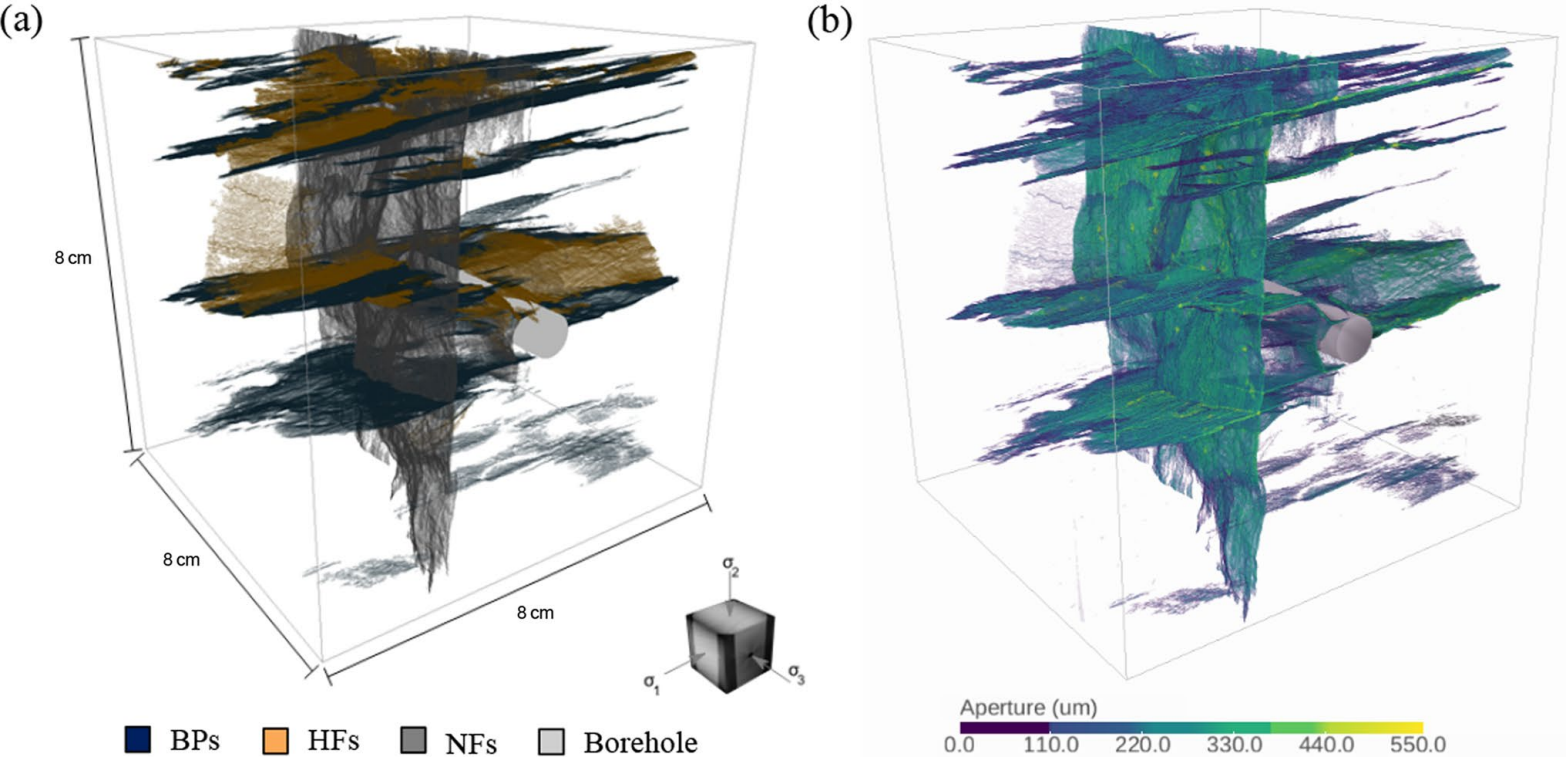
**a** 3D view of the fracture network differentiating the three categories of fractures by color (opened bedding planes in blue, opened natural fractures in grey, newly generated hydraulic fractures in orange) **b** 3D view of fracture aperture distribution. Refer to https://geogroup.utoronto.ca/li-et-al-2022/

The reconstructed fracture network of the tested sample (Fig. 19a) consists of three fracture sets: (1) bedding planes (BPs), (2) natural fractures (NFs), and (3) newly generated hydraulic fractures (HFs). In fact, as portrayed by the resulting fracture network, the wellbore seems to initiate a hydraulic fracture that opens against the intermediate principal stress prior to interacting with the pre-existing natural fracture and the bedding planes of the rock fabric. The geometry depicts that each fracture is non-planar and intersected with other fractures. In addition, the aperture of each individual fracture was measured to characterize the hydraulic conductivity in the following numerical simulation. For each fracture, we analyzed its aperture based on the 2D fracture slice along the fracture surface and counted the number of voxels in the direction normal to the 2D fracture on each slice. The obtained aperture shows heterogeneous characteristics (Fig. 19b).

### Seepage characteristics of the hydraulically fractured rock sample

**Fig. 20:**
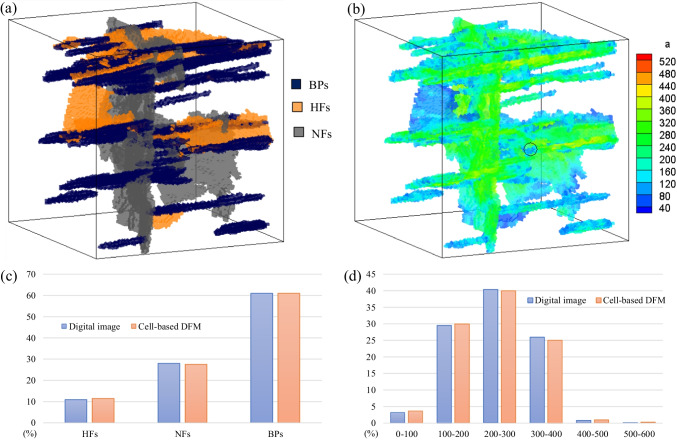
3D visualization of **a** Numerically reconstructed microfractures network and **b** Fracture aperture distribution (μm). The comparison of the **c** Fracture proportion and **d** Aperture distribution (μm) between the input digital image data and the numerically reconstructed fracture network using cell-based DFM

The 3D lab-scale hydraulically fractured shale sample is then reconstructed in the present numerical model to simulate the fluid flow characteristics during the production process. To maintain a high accuracy of numerical results with an acceptable computation cost, a 100 × 100 × 100 cell are set in dimension with a cell size of 0.8 mm. The reconstructed microfracture network skeleton according to the cell-based DFM is shown in Fig. 20a. The resulting numerical model effectively preserves the location and connectivity of the digitalized fracture networks even if the fracture pattern is extremely heterogeneous and complex. Fracture apertures are determined by the geometrical features of the fractures in the digital images (Fig. 20b). The quantitative comparison of the fracture proportion and aperture distribution between the input digital image data and the numerically reconstructed fracture network confirms the accuracy of the numerical model (Figs. 20c, d).

**Fig. 21:**
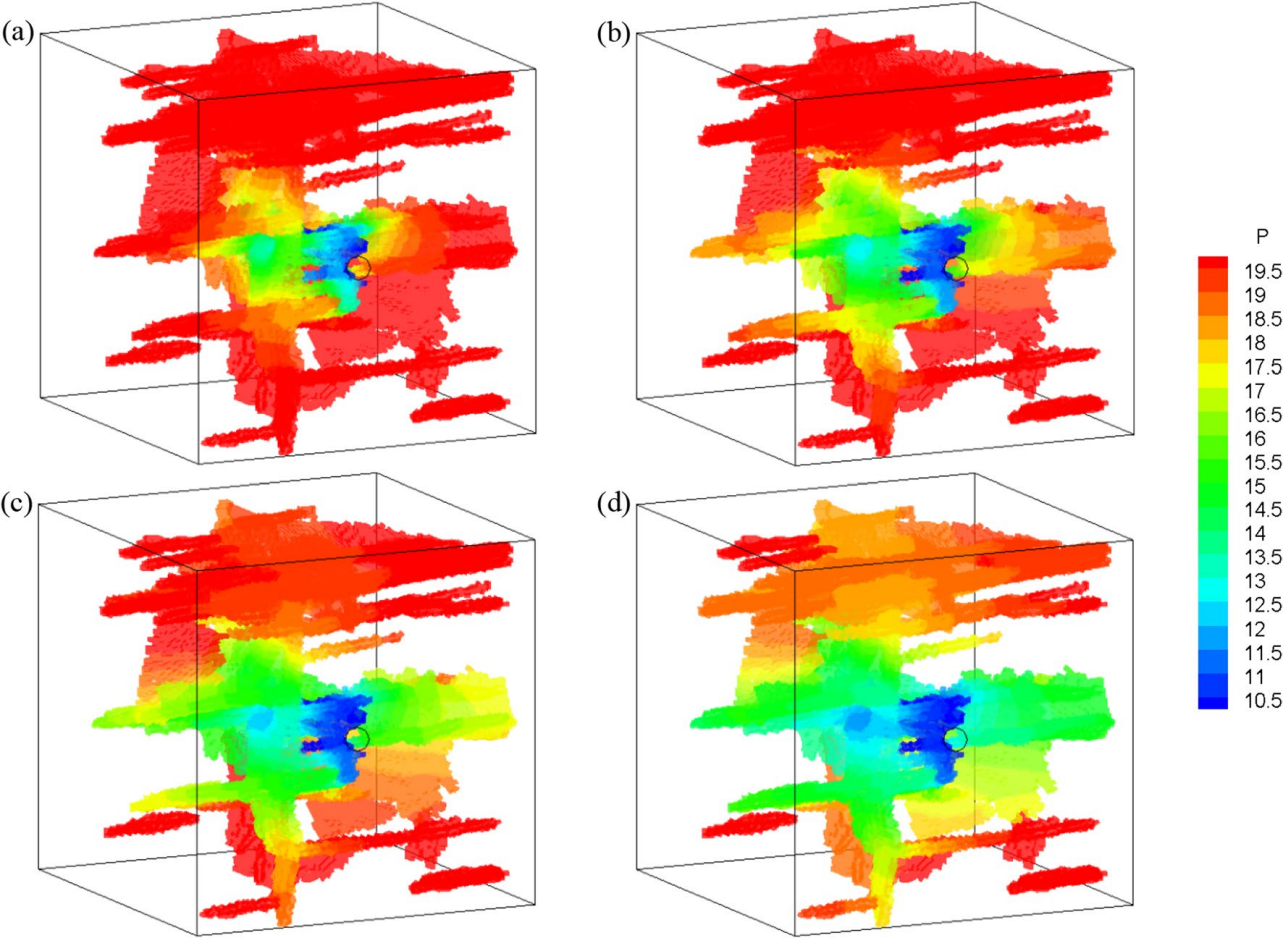
Fluid pressure distribution (kPa) in fracture networks at *t* = 10 ms, 20 ms, 50 ms and 200 ms

After the reconstruction of the fracture network, the seepage process is simulated. The initial pore pressure of both matrix and fractures are 20 MPa, with a pressure gradient of 10 kPa/m (Wozniakowska and Eaton 2020). A bottom hole pressure, *p*_w_ = 10 MPa is applied to simulate the production process. The fluid parameters are the same as those in Sect. 2.3.1, while the permeability of the rock matrix is *k* = 3 × 10^−16^ m^2^, the porosity of the matrix *ϕ* = 0.05, and the exchange coefficient is 10^−10^ m/(Pa s) (Vishkai et al. 2017; Vishkai and Gates 2019). The fluid pressure distribution during the seepage process is shown in Fig. 21. The fluid pressure of fractures directly connected to the wellbore decreases rapidly, while pressures of fractures far away from the wellbore (or indirectly connected to the wellbore) decrease at a slower rate. In fact, the pressure at the isolated fractures hardly changes. The simulation also shows that seepage in the microfracture network is strongly heterogeneous and anisotropic. Pressure varies rapidly in the natural fractures and bedding planes with larger apertures. However, fluid flows slower in the hydraulically induced fractures, which are far away from the wellbore and have smaller aperture.

**Fig. 22:**
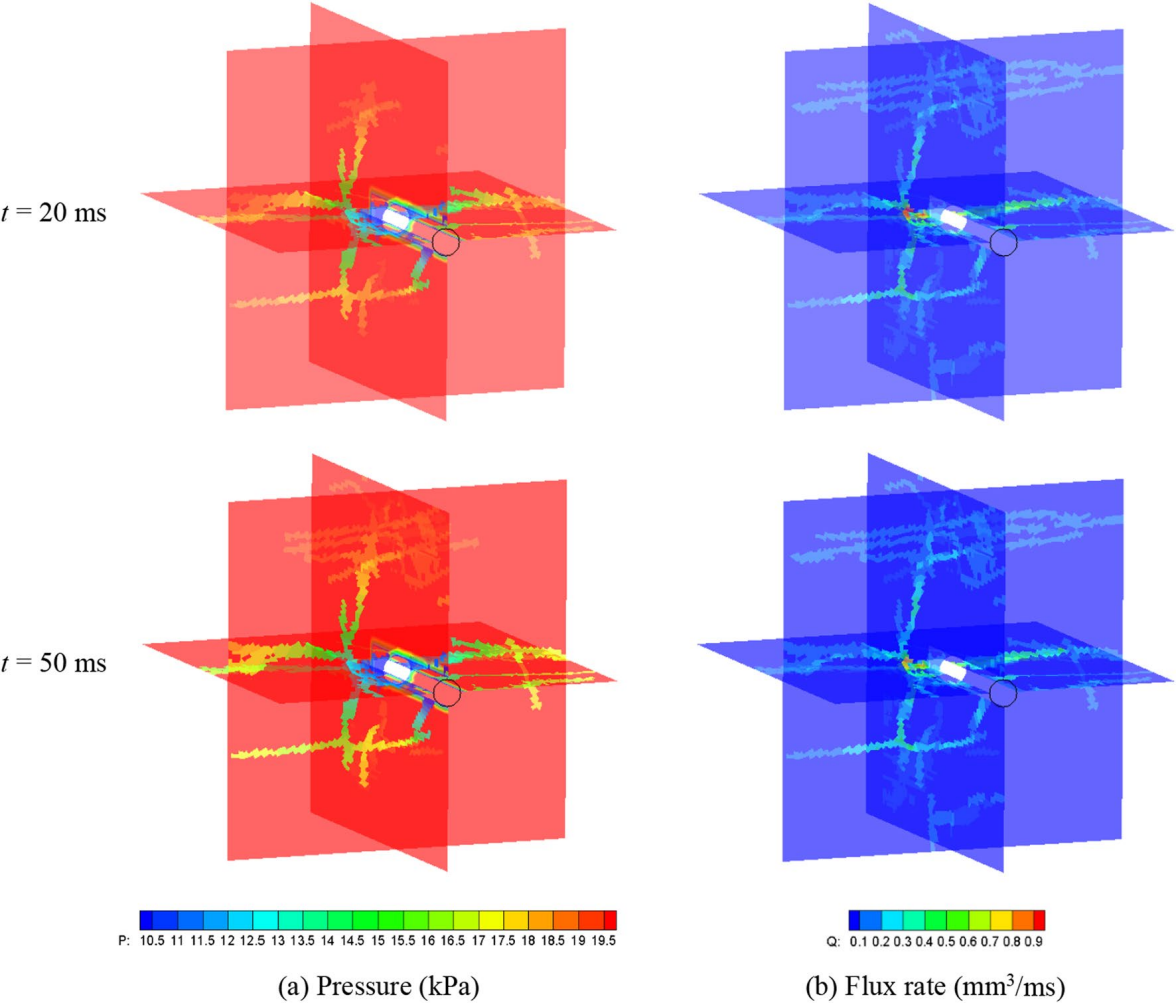
Sectional diagrams of **a** Pressure and **b** Flux rate distribution at *t* = 20 and 50 ms

Cross-section views at planes of *x* = 40 mm, *y* = 40 mm, and *z* = 30 mm of the fracture planes and rock matrix, are also obtained to show the pressure and fluid flux distribution (Fig. 22). The result shows that the highly permeable fractures severely distort the pressure distribution in the porous medium. Comparing the flux inside the porous matrix with the velocity field inside the fracture shows that the flow mainly occurs through the fractures. This indicates that the presence of fractures has a strong controlling effect on seepage, while the fluid flow in the matrix with very low permeability (orders of magnitudes lower than fracture permeability) can be virtually ignored. The pressure in fractures connected to the wellbore is lower than in fractures far away from the wellbore, and the fluid flux is also higher near to the wellbore. In addition, the pressure distribution and fluid flux distribution are not uniform, showing obvious heterogeneity.

**Fig. 23:**
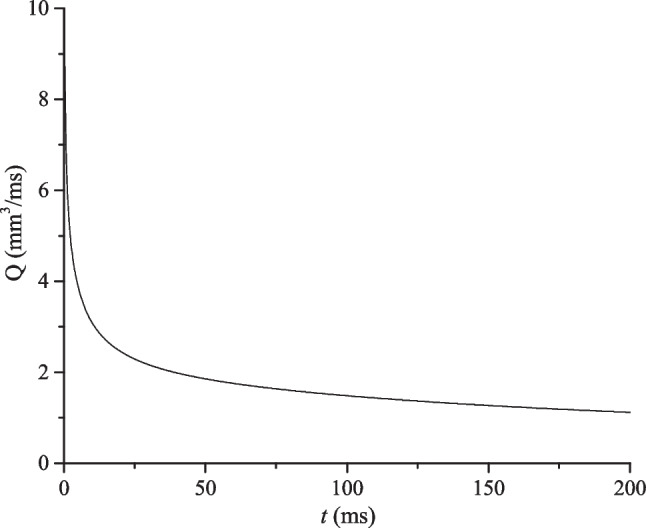
Production (total fluid) rate at the wellbore

During the initial post-treatment period the production rate for the modeled rock volume, at constant bottom hole pressure, quickly declines towards a steady value (Fig. 23). This can be explained by the fact that at the beginning of the production process the fluid flows mainly through the fractures, which causes a sudden drop in the well bore pressure. Then, as the pressure gradient decreases rapidly, the fluid flux drops, and the matrix starts to feed the fracture network at a lower rate function of the low matrix permeability. During this flow period the production rate at the wellbore becomes very low and pressure starts to drop slowly.

## Discussion

This paper proposed a novel framework for fluid flow simulation that can be applied directly to fractured porous medium extracted from digital images. The framework is conceptually simple and computationally efficient, however, the approach brings rise to some critical issues that should be further discussed.

### Effect of mesh size and ziggag approximation

**Fig. 24:**
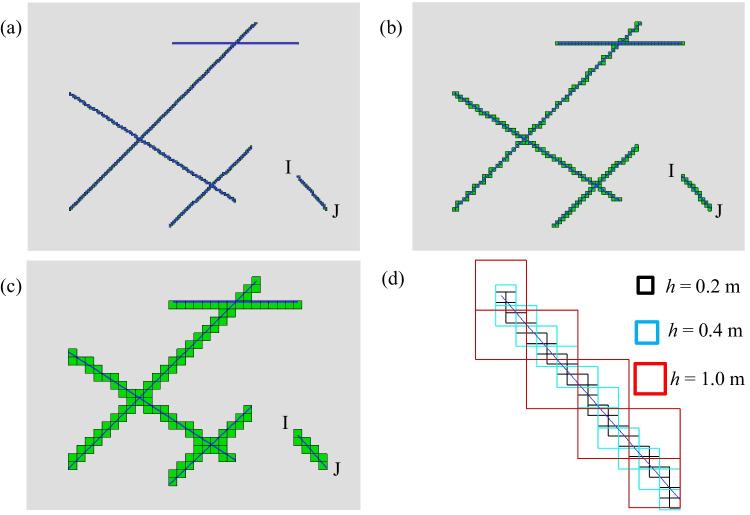
Zigzag fracture representation of validation test 3 (Sect. 2.3.3) with different mesh size **a** *h* = 0.2 m, **b** 0.4 m and **c** 1.0 m. The blue line represents the actual fracture shape, and the green cells represent the fracture elements. **d** A close view of the representation of an isolated fracture (IJ) with different mesh sizes

Fracture connectivity and local aperture have a direct impact on the permeability of fractured rocks; therefore, it is critical to capture these parameters accurately from the 3D images of the fractured rocks. In the present method, the reconstructed fracture network is generated based on pixel values of the digital image and a user-defined resolution. Section 2.3.2 proposed a simple case that investigated the mesh sensitivity (i.e., a user-defined resolution) for a single fracture, which shows that a finer mesh will give a more accurate solution with an error of less than 0.6%. However, for more complex fracture networks (i.e., Sect. 2.3.3), the “zigzag” approximation of the fracture geometry may occur (Fig. 24), which potentially has more significant effects on the fracture connectivity and accuracy of the results.

**Fig. 25:**
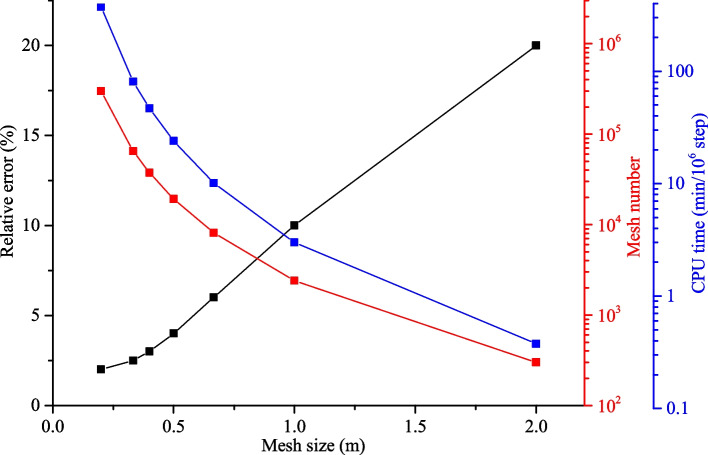
The relative error, total element numbers and CPU time with varying element size

To quantitively evaluate the effect of cell size, we analyzed the permeability and resistivity of models in Sect. 2.3.3 with varying mesh sizes (from *h* = 0.02 to 2 m). Figure 25 plots the relative error between numerical results and Yan’s solution (Yan et al. 2022) along monitoring line L_2_ when the element size varies. The relative error can be given as (Flemisch et al. 2018):

25 $$e_{\text{r}} = \sqrt {\frac{1}{n}\sum {\left( {\frac{{p_{i} - p}}{p}} \right)^{2} } }$$

where *p*_*i*_ is the simulated pressure in this model, *p* is the reference pressure, *n* is the total number of monitoring nodes along the monitoring line L_2_.

The results show that the mesh size affects the simulation accuracy, and the maximum difference is over 20% when the mesh size is relatively large (i.e., *h* = 2 m, corresponding to 300 elements). The relative error decreases when the element size decreases and is only 2% when the element size is less than 0.2 m (corresponding to 300,000 elements). It is evident that a finer element size provides a more accurate solution, however, the element number rapidly increases (almost a cubic relationship) with the decrease of element size (Fig. 25), which induces a heavy computation burden. The CPU time increases from 3 min to about 300 min every 10^6^ steps, when the mesh size decreases from 2 m to 0.2 m. In addition, when the element size decreases to the voxel size, the proposed method is degraded to the direct digital image volume method, which is extremely computationally expensive. All the numerical tests were performed on a PC with 2.67 GHz Intel-Corei7920 CPU and 8 GB of RAM.

### Pipe-network based cell-centered finite volume method

**Fig. 26:**
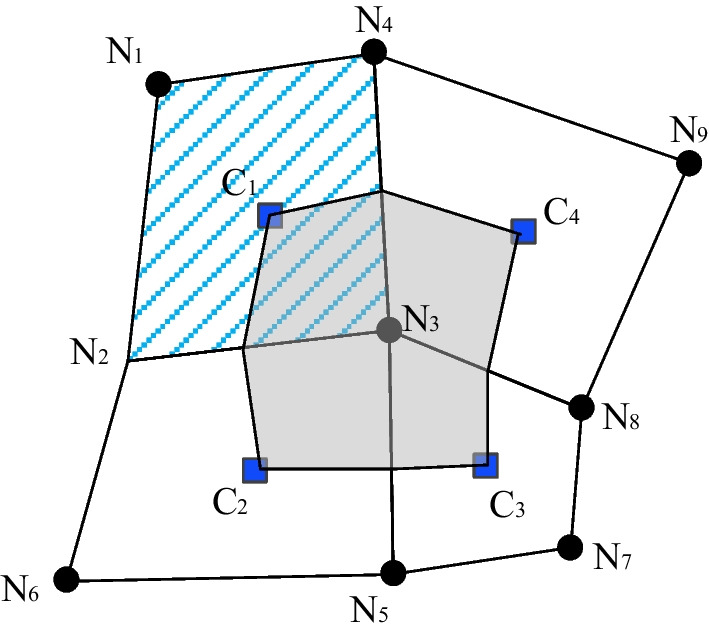
Control volume partitioning for cell-centered and node-centered finite volume discretization. N and C represent grid nodes and cell center, respectively. The control volume for a node-centered discretization around the grid node N_3_ is shaded. The control volume for a cell-centered discretization around the cell center C_3_ is hashed

The cell-centered finite volume method utilizes any given cell itself as the corresponding control volume and the information (e.g., mass, volume, pressure, flux) is stored at the cell center. Compared to the node-centered (also referred as vertex-centered) type (Fig. 26) (Ahn and Shashkov 2007; Diskin and Thomas 2011; Eymard et al. 2010), the cell-centered type is simpler and more efficient and is computational inexpensive, which is suitable for large-scale problems.

The pipe network model used in the finite volume method is conceptually simple, where fractures and porous media are connected via pipes in the domain space. Therefore, it is convenient to simulate flow in the continuous domain embedded with discontinuities. The 3D fluid flow problem is converted to equivalent 1D pipe flow, which significantly accelerates the calculating efficiency and significantly reduces computational time. Generalized transmissibility is proposed for fracture-fracture, fracture-matrix, and matrix–matrix entities connection, associated with different properties.

**Fig. 27:**
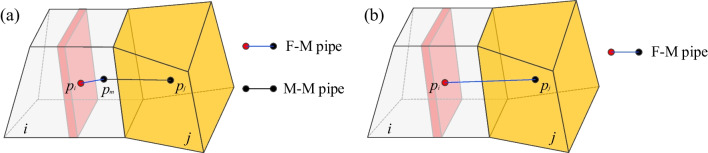
The exchange fluid flow in **a** Conventional DFM and **b** The present model

Especially, an explanation should be given for the treatment of the exchange flow. In reality, the fluid exchange should occur directly between the fracture and matrix in the fracture element (Fig. 27), which is widely used in conventional DFM (Rao et al. 2020; Xu et al. 2020). In the present method, the treatment of the fluid exchange is simplified as the fluid exchange between the fracture-cells and transition cells. The simplification is warranted for two main reasons: (1) the fracture plane is implicitly represented with the fracture-voxels, and the separated matrix cannot be determined; and (2) when the cell size is relatively small, the effect of the matrix flow in the fracture-cell can be ignored. Thus, the accuracy can be ensured with a simpler calculation algorithm, as proved in Sect. 2.3.3.

### Heterogeneity and the anisotropy feature of fluid flow

**Fig. 28:**
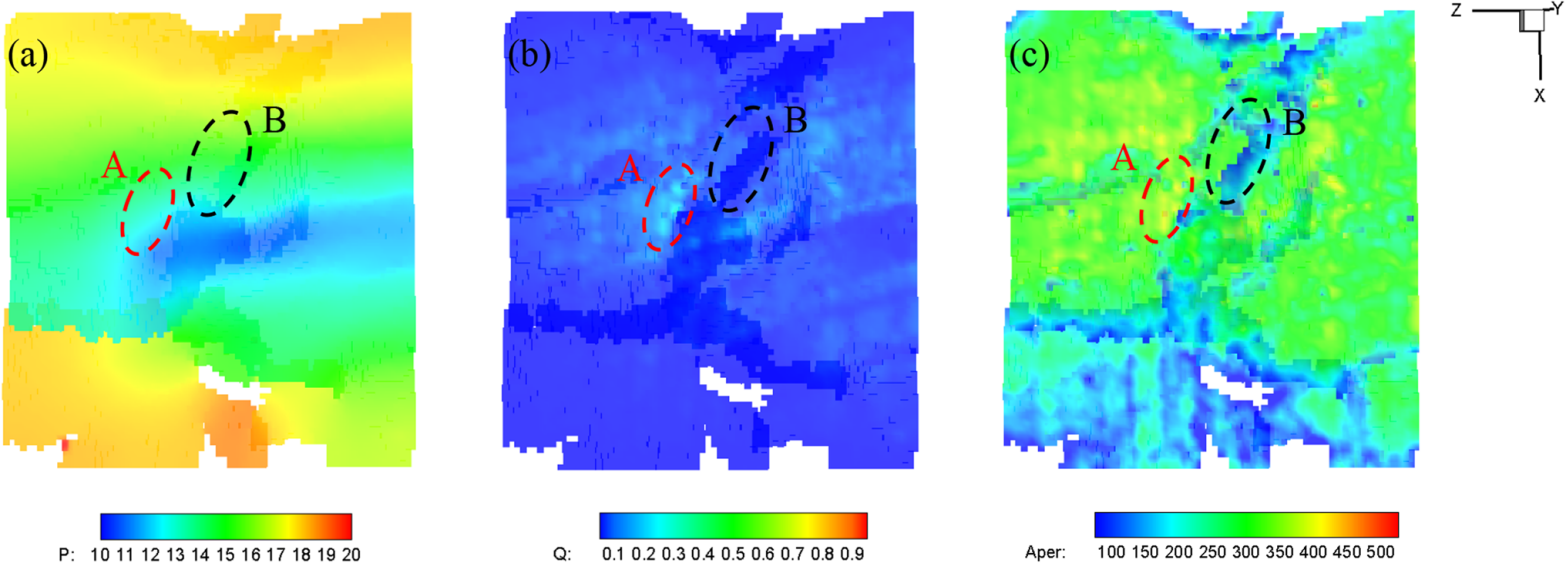
Heterogeneous fluid flow characteristics in a nature fracture in Sect. 3**a** Pressure (kPa), **b** flux (mm^3^/ms), and **c** Aperture distribution (μm). Zone A shows a highly conductive zone with large aperture, while Zone B represents the blocking zone with small aperture

In the present model, the fracture properties are extracted from the digital image, which presents better quantitative evaluation of the fracture geometrical features. The specifically assigned fracture aperture within each fracture segment allows the presented fracture model to capture the heterogeneity of the complex fracture geometry, resulting in a more realistic fluid flow calculation. For example, as shown in Fig. 28, the heterogeneous fluid flow characteristic in a nature fracture (Sect. 3) is observed, where fractures with particularly high or low permeability can act as flow conduits or barriers, respectively.

The labels of fracture-cells represent the connectivity between fracture segments. Thus, permeability between connected fracture cells is calculated according to the cubic law, which is higher than the permeability in other directions. Particularly, when multiple cracks with varying apertures intersect within one element. The specific aperture in different fracture sets can well simulate the heterogeneity and the anisotropy feature of the local permeability, where fluid tends to flow along fractures with higher permeability. In addition, the virtual aperture is independent of the mesh size, which can accurately simulate the fluid flux within a relatively large mesh.

## Conclusions

Accurately simulating the fluid flow in the real fracture network is important to better understand the fluid behavior in many underground engineering. This work proposes a novel simple numerical framework for the flow simulation in realistic fractured porous media obtained by 3D high-resolution images, aiming at both high accuracy and computational efficiency. The main conclusions can be drawn as follows:

1. The cell-based discrete fracture-matrix model (DFM) can well reconstruct fractured rock with complex fracture geometries (e.g., tortuous features, variable fracture apertures, and complex fracture intersections). The implicit fracture apertures eliminate the difficulty in handling fracture and matrix of different scales.
2. A pipe-based cell-centered finite volume method is proposed to simulate flow in the complex fractured porous media (including matrix flow, fracture flow, as well as exchange flow). The performance of this model is validated against analytical/numerical solutions.
3. Although a finer mesh provides a more accurate solution, a moderate matrix grid block size (i.e., coarse cell) can also achieve a good balance between computational efficiency, flexibility and accuracy. This feature confirms the potential application of using the proposed method in the long-term simulation of large-scale problems.
4. The complex fracture networks control the fluid flow process, and the opened natural fractures behave as primary fluid pathways. Heterogeneous and anisotropic features of fluid flow, due to the varying aperture and the fracture connectivity, are well captured with the present model.

Accurate fracture network representation and high computational performance are the two major bottlenecks when it comes to fluid flow simulation in a fractured medium. Different scales of fractures, ranging from kilometers to millimeters, exist in many environmental and engineering applications (e.g., oil/gas production, geothermal exploration, CO_2_ geological sequestration, and nuclear waste disposal), which requires special attention when using the proposed cell-based discrete fracture-matrix flow model. In addition, this simple yet efficient framework of fluid flow simulation can be further extended to multiple phases (e.g., air/water/oil) and multiple field (thermo-hydro-mechanical) applications, which is the aim of our future work.

## Data Availability

The detailed information of the 3D fracture network (coordinates and aperture) of the hydraulically fractured rock sample (Sect. 3) is available at https://geogroup.utoronto.ca/li-et-al-2022/. For other data availability, please contact the corresponding author.
